# Disrupting emotional memory: PASER as a potential reconsolidation-based intervention in stressful experiences

**DOI:** 10.3389/fcogn.2026.1868072

**Published:** 2026-08-07

**Authors:** Jonathan Emanuel Petrone, Lucía Daniela Romero, Antonella Oliva Montaudo, Lorna A. Evans, Diego Raúl Piñeyro

**Affiliations:** 1 Faculty of Psychology, University of Buenos Aires, Buenos Aires, Argentina; 2 Neuroscience and Artificial Intelligence Research Laboratory of the Army Faculty, National Defense University, Buenos Aires, Argentina; 3 Procesamiento Asistido en Situaciones Estresantes Recientes Mental Health Association, Buenos Aires, Argentina; 4 Faculty of Psychology and Human Relations, Universidad Abierta Interamericana, Buenos Aires, Argentina; 5 Faculty of Psychology and Psychopedagogy, University of Salvador, Buenos Aires, Argentina; 6 Aerospace and Aviation Medicine, Department of Internal Medicine, Mayo Clinic, Jacksonville, FL, United States; 7 Latin American Aerospace Medicine, Engineering and Biotechnology Association, Jacksonville, FL, United States

**Keywords:** desensitization, emotional disturbance, memory reconsolidation, PASER, psychological first aid

## Abstract

**Introduction:**

Stressful experiences may generate persistent emotional distress that could interfere with daily life. Early intervention is key to reducing those emotional disturbances and potentially preventing the development of more severe pyschopathology. The present research consists of two studies that evaluate the effects of the PASER technique on levels of emotional disturbance.

**Methodology:**

In study 1, a quasi-experimental repeated-measures within-group design was used, with four assessments conducted through the APEEM inventory in relation to a non-pathological stressful situation selected by each participant. The first three measurements were mediated solely by brief imaginal exposure (inherent to the APEEM instrument) in order to control for the cumulative effects of exposure and habituation over time. Following each of these assessments, a 15-min distractor task was administered. The fourth measurement was conducted after the group application of the PASER technique. In study 2, an experimental design was conducted, assessing the effects of the variables “measurement time point” (baseline and post-test), “sex” and “intervention condition” (control vs. PASER).

**Results:**

The results showed a progressive reduction in emotional disturbance as time progressed in both studies. Participants receiving PASER exhibited a significantly greater reduction in emotional disturbance than those in the control condition. These findings support the effectiveness of PASER in reducing emotional disturbance beyond the effects attributable to repeated exposure and the passage of time.

**Conclusions:**

These results suggest that PASER produces an effect greater than simple exposure-based desensitization, potentially promoting memory reconsolidation processes. Longitudinal studies are recommended to confirm the stability of these effects.

## Introduction

Stressful experiences are an inherent part of human life and vary considerably in intensity, duration, and personal significance. Although many of these experiences may not meet the diagnostic criteria for psychological trauma, they may nevertheless generate persistent emotional distress when recalled, influencing wellbeing, emotional regulation, cognitive performance and decision-making. Consequently, understanding how stressful memories are encoded, stored, and later retrieved has become an important area of research in both clinical and non-clinical populations.

Current evidence suggests that the emotional impact of a stressful event depends not only on the characteristics of the event itself but also on the way in which the experience is processed and integrated into autobiographical memory. During the hours immediately following an emotionally significant experience, memory traces remain dynamic and susceptible to modification. This period offers a valuable opportunity for interventions aimed at facilitating adaptive processing of the experience and reducing the emotional disturbance associated with its later recall. Such interventions may be particularly relevant in high-performance occupations characterized by frequent exposure to critical incidents, including police officers, military personnel, emergency medical professionals, rescue workers and air traffic controllers, where emotional interference may affect both psychological wellbeing and operational effectiveness (Azzara et al., 2018; Azzollini et al., 2015; Benedek et al., 2007; Everly and Flynn, 2006; Piñeyro and Azzollini, 2019).

Among the most commonly used early interventions are defusing and debriefing protocols, which seek to reduce emotional distress through guided recollection and discussion of the stressful event. However, evidence regarding their effectiveness has been mixed (Benedek et al., 2007; Bisson et al., 2008; Hobfoll et al., 2007; McNally et al., 2003; Ruzek et al., 2007). Some studies suggest that intense emotional reactivation shortly after the event may not always promote adaptive processing and, under certain circumstances, could strengthen distressing emotional responses (Farchi et al., 2014; Svetlitzky et al., 2020). Given that memory traces remain highly labile during the early post-event period, interventions that excessively increase emotional arousal may inadvertently contribute to maladaptive processing of the experience (Piñeyro, 2022; Piñeyro and Azzollini, 2022b).

In this context, PASER (which stands for “Procesamiento Asistido en Situaciones Estresantes Recientes”, translated as “Assisted Processing for Recent Stressful Situations”) has been developed as a brief intervention specifically designed to facilitate the adaptive processing of recent stressful experiences (Cattaneo et al., 2025; Piñeyro et al., 2017; Azzara et al., 2018; Piñeyro et al., 2024). Its application requires approximately 10–15 mins and can be implemented in operational settings such as evacuation centers, fire stations, field hospitals, military deployments, or air traffic control facilities. Unlike traditional exposure-based approaches that primarily seek habituation through repeated recall of the stressful experience (Caballo, 1997; Caballo and Mateos Vilchez, 2000; Foa and Kozak, 1986; Olivares Rodríguez and Méndez Carrillo, 2014), PASER aims to facilitate adaptive memory reprocessing and integration into autobiographical memory, reducing the emotional intensity associated with subsequent recall. To achieve this objective, PASER incorporates elements derived from evidence-based interventions, including cognitive restructuring, alternating bilateral neurostimulation, and autobiographical narrative reorganization, which may contribute to modifying the emotional meaning assigned to the remembered event (Piñeyro et al., 2024).

The theoretical foundation of PASER is grounded in contemporary models of memory reconsolidation. Research has shown that the retrieval of a recently acquired memory can temporarily destabilize its neural representation, creating a window during which new information may be incorporated before the memory is stored again (Coccoz et al., 2013; Delorenzi et al., 2014; Elsey and Kindt, 2017; Kindt and Van Emmerik, 2016; Larrosa et al., 2017). Interventions delivered during this period may facilitate the integration of adaptive interpretations and meanings into the memory trace, thereby reducing the emotional disturbance associated with future retrieval. Rather than focusing exclusively on the prevention of psychopathology, this perspective emphasizes the possibility of promoting more adaptive memory representations and improving emotional adjustment following stressful experiences.

From a cognitive standpoint, PASER relies heavily on working memory processes and directed attention. Through the coordinated use of attentional tasks, motor activity, bilateral stimulation, and guided cognitive processing, the intervention may facilitate the organization of the stressful experience into a more coherent and integrated autobiographical representation (Masin et al., 2025). This process may also reduce proactive interference from pre-existing maladaptive schemas or interpretations that could otherwise influence the appraisal of the event (Engle et al., 1999; Eysenck et al., 2007; Friedman and Miyake, 2004; Kane and Engle, 2000; Macizo et al., 2006; Piñeyro and Azzollini, 2022b; Soriano et al., 2004). In this sense, the facilitator's instructions serve not only to guide attention but also to promote adaptive emotional regulation and meaning-making.

Although preliminary studies have reported favorable outcomes following PASER implementation in emergency response, rescue, security, and defense personnel (Piñeyro and Azzollini, 2022a; Azzollini et al., 2017; Piñeyro et al., 2024; Cattaneo et al., 2025; Masin et al., 2025; Romero et al., 2025), its mechanisms of action remain insufficiently explored. In particular, little is known about the extent to which reductions in emotional disturbance result from the PASER intervention itself or from the repeated retrieval of the stressful memory. Furthermore, evidence regarding its application in non-operational and non-clinical populations remains limited.

Therefore, the following two studies were designed to examine the desensitization effects of the PASER technique. In Study 1, a quasi-experimental repeated-measures design with four assessment points was employed. Changes in emotional disturbance associated with stressful autobiographical memories were examined following repeated memory recall and the application of the PASER technique in a sample of university students. Emotional disturbance was assessed using the APEEM inventory across four successive stages of memory retrieval to determine whether the pattern of change observed during repeated imaginal exposure differed following the application of the PASER technique. Study 2 employed an experimental design with random assignment of participants to either a control group or an experimental group in order to address the limitations of Study 1. These limitations were mainly related to the absence of a control group, as Study 1 employed an exploratory repeated-measures design on the same sample. In addition, the potential influence of the variable “sex” on treatment outcomes was not examined in Study 1 and was, therefore, also analyzed in Study 2.

## Study 1

### Objective

The objective of the present study was to evaluate differences between emotional disturbance levels observed across repeated imaginal exposure and those observed following the PASER intervention in a sample of university students.

### Hypothesis

The pattern of emotional disturbance was expected to show a greater reduction following the application of the PASER technique that the pattern observed across repeated imaginal exposure.

### Methodology

#### Design

This is a quasi-experimental study (Pereda Marín, 1987) with a repeated-measures design, including four assessment points of emotional disturbance levels related to a recent stressful event. This design allows the examination of changes in emotional disturbance across repeated memory retrieval, as well as whether the pattern of change differed following the application of the PASER technique.

#### Participants

The sample consisted of 67 university students from the Faculty of Psychology at the University of Buenos Aires, enrolled in the Therapeutic Support Diploma Program. Gender distribution was 53 women (79.1%), 12 men (17.9%), and 2 participants who did not specify their gender (3%). Participants' ages ranged from 20 to 55 years (*M* = 25.84; *SD* = 7.65).

#### Instruments

To assess emotional disturbance, the Emotional State Self-Perception Inventory was used (hereafter referred to by its Spanish acronym, APEEM; *Test de* “*Autopercepción del Estado Emocional*”). This instrument consists of seven items that assess emotional and physiological reactions on a Likert-type scale ranging from 0 (“absence of affectation”) to 10 (“maximum affectation”) in relation to a recent and non-pathological stressful situation chosen by each participant that had occurred within the previous 3 weeks.

The instrument has demonstrated high internal consistency (Cronbach's α = 0.88) and construct validity confirmed through exploratory and confirmatory factor analyses (Piñeyro et al., 2019; Piñeyro and Azzollini, 2022a). (Piñeyro, 2018; Piñeyro et al., 2019; Piñeyro and Azzollini, 2016).

The APEEM administration procedure requires participants to close their eyes and imaginatively connect with the recent stressful situation they wish to evaluate, evoking images, sounds, smells, and other related sensations. This initial step generates a brief exposure of approximately 30 s to the situation before responding to the items.

Additionally, as a group psychological intervention instrument, the PASER protocol was used. This is a brief technique designed to be applied in emergency contexts or during the first hours following a stressful event. Lasting approximately 15 mins, PASER includes three main components: (a) induction of a state of relaxation and bodily connection (Jacobson, 1929), (b) controlled visualization of the stressful situation from a distanced perspective and organized temporally, and (c) introduction of adaptive meanings and resources through the facilitator's verbal instructions, including statements such as: “*we realize that we are not alone... that we can always count on someone... and we propose to improve and change those things we can change to feel better... and little by little accept those others that we cannot change... that are part of life, to continue moving forward with our lives, our projects... because we deserve it*.” After this closing statement, the facilitator informs participants about the possible bodily sensations and emotional experiences they may notice following the intervention.

The technique is based on principles of exposure-based desensitization, bilateral stimulation, and emotional memory reconsolidation. Previous studies have documented its effectiveness in reducing emotional disturbance in both general populations and emergency personnel (Piñeyro et al., 2024; Cattaneo et al., 2025; Masin et al., 2025; Romero et al., 2025).

It should be noted that, regardless of the fact that each participant selects a particular memory whose specific content is unknown to the facilitator, the procedural guidelines of the technique ensure that cognitive intrusions do not divert the course of processing. The design of the intervention provides a structured framework that guides the flow of processing, preventing intrusive thoughts from directing the experience in a dysfunctional manner. To this end, the technique proposes a temporally organized reprocessing procedure aimed at regulating anxiety levels and promoting positive cognitions related to self-esteem. For example, by instructing participants to visualize the stressful situation on a movie screen (observing its beginning, development, and ending as though watching a film) the technique induces a shift toward a third-person perspective using explicit temporal cues. After alternating with a safe-place exercise to reduce emotional activation, a second visualization of the movie employs fast-forward imagery and blurred perceptual representations to attenuate the intensity of the memory. Subsequently, the use of a blank screen functions as a thought-control mechanism that blocks further intrusive thoughts. Finally, the exercise concludes by fostering positive schemas focused on perceived social support, self-efficacy to modify controllable aspects of the situation, and acceptance of those aspects of the experience that cannot be changed.

#### Procedure

During a university class, participants were instructed to choose a recent stressful situation (occurring within the previous 3 weeks) or a situation that caused concern due to its possible occurrence. Participants were instructed not to work on situations related to any clinical diagnosis or issues considered psychopathological. At the same time, they were asked to work with the same stressful scene during each assessment point, without changing it.

Once each participant selected the stressful situation, the level of emotional disturbance was assessed using the APEEM inventory at three different time points separated by 15-min intervals, during which a lecture on theoretical concepts of psychological first aid applied to rescue workers was provided. It should be clarified that the APEEM instrument requires the participant to visualize for a few seconds the memory intended to be measured.

In this way, it was possible to establish a baseline of emotional disturbance (APEEM1) and an estimation of the effects of emotional disturbance reduction produced by the simple passage of time and the imaginal exposure generated by the instrument itself (APEEM2 and APEEM3; habituation and desensitization control). Subsequently, the PASER technique was applied to the entire sample (a protocol lasting approximately 15 mins), in order to obtain a final post-intervention measurement (APEEM4).

Therefore, the measurements analyzed in this study were as follows:

- APEEM 1: Baseline assessment of emotional disturbance in response to the chosen scene.
- APEEM 2: Assessment conducted 10 mins later, with no intervention, reflecting only the effects of time and repeated memory recall.
- APEEM 3: Assessment conducted 10 mins after APEEM 2, again with no intervention.
- PASER intervention: Application of the PASER protocol (approximately 15 mins).
- APEEM 4: Post-intervention assessment conducted immediately after the PASER intervention to evaluate changes in emotional disturbance.

#### Data analysis

Data were analyzed using SPSS v26, employing descriptive statistics (Table 1), the Kolmogorov–Smirnov normality test, and repeated-measures analysis of variance (RM-ANOVA) with Huynh–Feldt correction to adjust degrees of freedom due to a violation of the sphericity assumption. At the same time, Bonferroni adjustment was used for *post-hoc* comparisons between measurements.

**Table 1:** Descriptive statistics (Study 1).

Measurement time	*N*	Minimum	Maximum	Mean	Std. deviation	Kurtosis	
	Statistic	Statistic	Statistic	Statistic	Statistic	Statistic	Std. error
APEEM 1	67	3	65	38.88	13.808	0.194	0.578
APEEM 2	67	1	68	36.37	15.185	−0.418	0.578
APEEM 3	67	0	61	31.36	15.324	−0.863	0.578
APEEM 4	67	0	55	17.96	11.729	0.710	0.578
Valid *N* (listwise)	67						

### Results

The assumption of normality was evaluated using the Kolmogorov–Smirnov test for each of the four measurement points. The results indicated that the three measurements prior to the PASER intervention did not show significant deviations from normality (*p* > 0.05). However, the fourth measurement, corresponding to the post-intervention assessment, showed a significant deviation from normality (*p* < 0.05). Descriptive statistics of the APEEM are displayed in Table 1.

Subsequently, the assumption of sphericity was assessed using Mauchly's test, which was significant (*W* = 0.61, *p* < 0.001), indicating a violation of this assumption. Therefore, the Huynh–Feldt correction (ε = 0.80) was applied to adjust the degrees of freedom.

**Figure 1:**
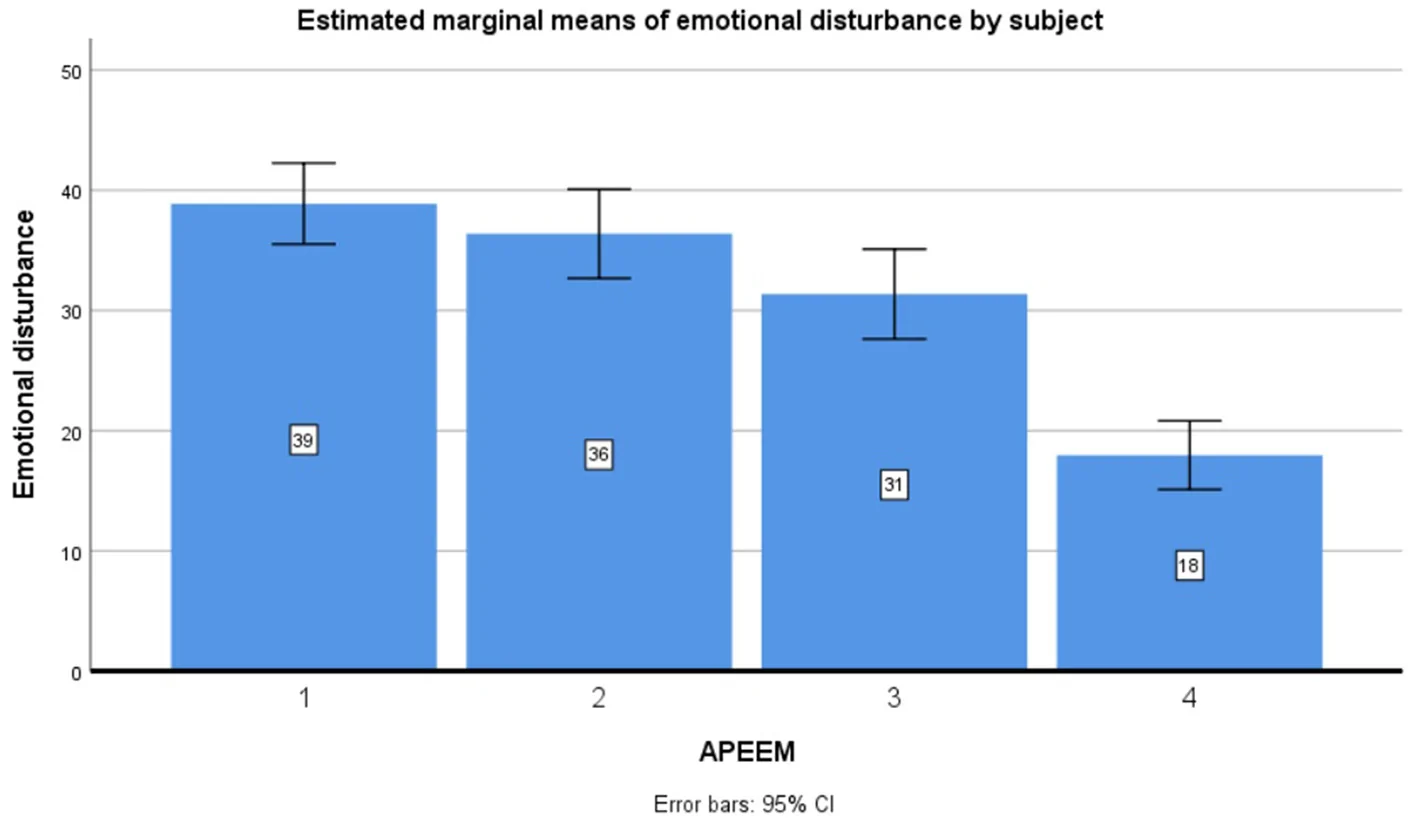
Estimated marginal means of emotional disturbance by subject in each of the four APEEM measurements.

The repeated-measures ANOVA showed a significant main effect of time, with Huynh–Feldt correction, *F*_(3, 64)_ = 86.685, *p* < 0.001, *ηp*^2^ = 0.803, indicating significant differences in emotional disturbance levels across the measurements. *Post-hoc* comparisons with Bonferroni adjustment revealed significant differences between all pairs of measurements: APEEM1 vs. APEEM2 (*p* = 0.011), APEEM2 vs. APEEM3 (*p* < 0.001), and APEEM3 vs. APEEM4 (*p* < 0.001; Figure 1). However, pairwise comparisons revealed a small effect size between APEEM 1 and APEEM 2 (*d* = 0.17), a small effect size between APEEM 2 and APEEM 3 (*d* = 0.33), and a large effect size between APEEM 3 and APEEM 4 (*d* = 0.98).

These results indicate that significant changes occurred in the values of each new measurement, suggesting sustained emotional improvement over time, with the greatest variation in scores occurring following the implementation of the intervention.

Because all *post-hoc* comparisons showed statistical significance, change scores (differences in change) among the four APEEM measurements were analyzed as follows:

- **Time 1:** refers to the within-subject change difference between APEEM1 and APEEM2 (APEEM2 – APEEM1);
- **Time 2:** refers to the within-subject change difference between APEEM2 and APEEM3 (APEEM3 – APEEM2);
- **Time 3:** refers to the within-subject change difference between APEEM3 and APEEM4 (APEEM4 – APEEM3).

A repeated-measures ANOVA was conducted on the change scores, which showed a significant interaction with Mauchly's sphericity assumption met (*W* = 0.948, *p* = 0.177), *F*_(2, 65)_ = 28.056, *p* < 0.001, *ηp*^2^ = 0.463. *Post-hoc* comparisons with Bonferroni adjustment revealed that the differences between Time 1 and Time 2 were not significant (*p* = 0.142), whereas the differences between Time 2 and Time 3 (*p* < 0.001) and between Time 1 and Time 3 (*p* < 0.001) were significant.

This indicates that the reduction in emotional disturbance levels between APEEM1 and APEEM2 (Time 1) was not significantly different from that achieved between APEEM2 and APEEM3 (Time 2). However, the reduction achieved between APEEM2 and APEEM3 (Time 2) and between APEEM3 and APEEM4 (Time 3) was significantly different, as was the reduction between APEEM1 and APEEM2 (Time 1) compared with APEEM3 and APEEM4 (Time 3).

**Figure 2:**
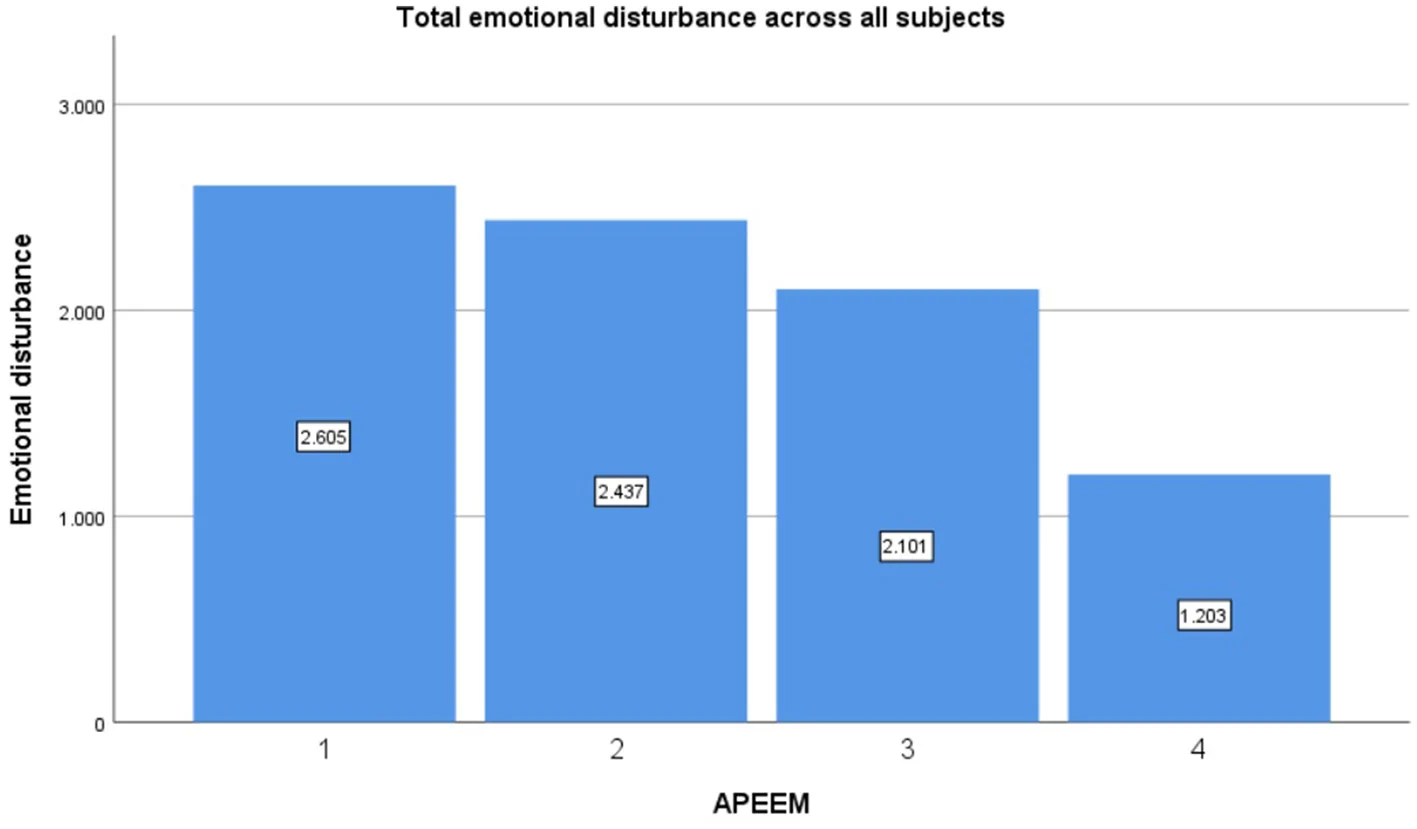
Total emotional disturbance across all subjects in each of the four APEEM measurements.

In summary, while slight reductions in emotional disturbance were observed during the first three measurements (attributed to repeated exposure), the most pronounced and significant reduction occurred after the application of PASER (Figures 1, 2).

### Discussion (study 1)

The results of the present study indicate that the PASER technique may be associated with a greater reduction in emotional disturbance than that observed through simple repeated exposure to the same stressful scene. This finding is consistent with previous research in which PASER has shown effectiveness in modulating emotional responses in populations exposed to recent highly stressful events (Piñeyro et al., 2023a,b, 2024; Cattaneo et al., 2025; Romero et al., 2025).

It is worth considering that, technically, the first measurement point is not a “pre-test” free of intervention, but rather the first post-initial exposure measurement, and interpretations should therefore be understood as relative comparisons between phases. The analysis using *change scores* (differences in raw scores between time points) constitutes a valuable contribution. This procedure, complementary to the original ANOVA, allows for direct comparison of the *magnitude of change* between intervals and demonstrates that the phase mediated by PASER generated a significantly greater reduction in emotional disturbance.

The pattern observed (progressive reductions across the first three measurements and a substantial decrease following the application of PASER) suggests that the change produced by the technique exceeds the exposure-based desensitization phenomenon described in the behavioral literature (Bouton, 2004; Foa and Kozak, 1986; Olivares Rodríguez and Méndez Carrillo, 2014; Wolpe, 1958). Exposure-based desensitization, as well as the passage of time, typically generate partial habituation to the mental re-experiencing of the scene; however, in this case, the post-intervention decrease following PASER was considerable after the three previous exposure instances, which points to the presence of additional mechanisms.

These observations are consistent with the preliminary findings reported in previous studies on the PASER technique. However, it should be noted that those studies presented methodological limitations that the present design sought to address. Specifically, although reductions in emotional disturbance have previously been reported, earlier investigations either lacked a control group (Masin et al., 2025), were limited in their external validity due to the use of substantially small samples (Cattaneo et al., 2025), did not include equivalent control groups (Piñeyro et al., 2024), or relied on pre-experimental designs with exclusively within-group pre–post comparisons (Romero et al., 2025). The present study extends these findings by using a repeated-measures design that explicitly examined the pattern of emotional change across multiple assessments prior to the PASER intervention. This approach allowed us to estimate the reduction attributable to repeated memory retrieval and the passage of time, showing that the decrease observed after PASER exceeded the changes associated with repeated imaginal exposure alone.

The sequential application of the PASER protocol steps includes elements that may induce the “surprise effect” necessary to activate the *labilization of neural engrams* associated with the stressful or concerning situation, facilitating their modification during the reconsolidation window (Coccoz et al., 2013; Delorenzi et al., 2014; Holmes et al., 2007; Kindt and Van Emmerik, 2016; Larrosa et al., 2017; Piñeyro and Azzollini, 2022b; Piñeyro et al., 2024).

The modifications produced through PASER would not be limited to reducing physiological and cognitive reactivity, as evidenced by APEEM measurements, but would also promote *adaptive restructuring* (Beck and Freeman, 1995; Clark and Beck, 2012) of the mental representation of the event. It is proposed that re-experiencing the memory with low levels of anxiety achieved through the initial relaxation phase of the technique contributes to this process.

Likewise, the shift in memory perspective from first-person to third-person may also provide novel (unexpected) elements that generate a new sense of control and safety, similar to the phenomenological aspects observed when *imagery rescripting* protocols are applied (Arntz, 2012; Holmes et al., 2007; Wild and Clark, 2011).

Additionally, the instructions provided by the facilitator administering the technique function as an External Central Executive (Baddeley, 1996; Baddeley and Hitch, 1974; Friedman and Miyake, 2004), transmitting support, expectations of possible change, and acceptance of unavoidable realities, thereby introducing new meanings into semantic and autobiographical memory (Masin et al., 2025).

These internal processes may have therapeutic effects as part of reconsolidation. However, even if functionally adaptive emotional outcomes are observed in relation to the lived experience, and although these outcomes could be due to memory reconsolidation processes, it should be noted that such processes are not directly quantifiable nor necessarily the primary objective of applying a desensitization technique, as reconsolidation remains a hypothetical construct (Kindt and Elsey, 2023).

The presence of memory reconsolidation is hypothetically inferred from the observed reduction in emotional disturbance; therefore, this process is experimentally manipulated indirectly through desensitization techniques. According to some authors, memory reconsolidation could be verified in longitudinal studies in which reductions in emotional disturbance scores remained stable even 1 year after the application of such techniques (Filmer et al., 2022; Peters et al., 2025).

From a neuropsychological perspective, the use of *bilateral neurostimulation*, characteristic of the “butterfly technique” performed with the hands during PASER, promotes the simultaneous activation of cortical and subcortical networks in both hemispheres, which may facilitate integration between perceptual and linguistic representations (Brewin, 2014; Brewin et al., 1996; Shapiro, 2004).

This multiformat processing is reinforced by the induced *shift in perspective* (third-person visualization and temporal organization of the scene), which increases emotional distancing, promotes cognitive restructuring, and reorganizes information in episodic, autobiographical, semantic, and perceptual memory, optimizing its storage in a less disturbing format and integrating emotional and cognitive changes (Brewin et al., 1996; Brewin and Smart, 2005; Piñeyro et al., 2024; Tulving, 1983, 1993).

### Limitations

This study presents limitations derived from its quasi-experimental design, including the absence of an equivalent control group and the lack of random assignment, which restrict its internal validity. Therefore, our next study incorporated an active control group, random assignment and the inclusion of Sex as an additional factor in the analysis.

## Study 2

### Objective

Considering the findings and limitations of Study 1, a second study with an experimental design was conducted to evaluate the interactions between the variables “measurement time point” (pre-test vs. post-test), “sex” and “intervention condition” (control vs. PASER).

### Hypothesis

Emotional disturbance levels in the experimental group were expected to show a greater reduction following the PASER intervention compared with those in the control group, which performed a distractor task.

### Methodology

#### Design

Through simple random assignment, participants were distributed into two groups: an experimental group (*n* = 26) and a control group (*n* = 25). However, three participants from the control group had to be excluded from the analysis due to incomplete data (blank responses or double markings on the measurement instrument), resulting in a final sample of 48 participants (experimental group: *n* = 26; control group: *n* = 22).

A 2 × 2 mixed factorial design was employed, with one between-subjects factor (Intervention Condition: PASER vs. control) and one within-subjects factor (Time: pre-test vs. post-test). The within-subjects factor operationalizes the temporal effect: the variation in emotional disturbance levels between the first and second exposure to the scene (10 mins later), regardless of the type of intervention. This factor makes it possible to isolate the potential desensitization effect due to repeated exposure (expected even in the absence of a technique) from the specific effect attributable to PASER.

#### Participants

The sample initially consisted of 51 students from the Faculty of Psychology at the University of Buenos Aires who participated voluntarily. All participants signed an informed consent form prior to their inclusion in the study. Three participants were excluded from the sample due to erroneous or incomplete completion of the assessment inventories. The final sample consisted of 48 participants, with a mean age of 24 years, of whom 83.3% were women and 16.7% were men.

#### Instruments

The Emotional State Self-Perception Instrument (APEEM) was used to measure the dependent variable “emotional disturbance.” It was administered at two time points: pre-test (baseline) and post-test (following the PASER intervention or the control task).

Additionally, the PASER protocol was used as the psychological intervention for the experimental group.

For the control group, a visual puzzle consisting of identifying the number of triangles contained within a composite geometric figure was used as the distractor task.

#### Procedure

The study was conducted in group sessions. At the beginning, all participants were asked to identify a recent personal memory of an event that had occurred within the last few weeks and that generated some level of anxiety, stress, or concern. Subsequently, the APEEM was administered to record the level of emotional disturbance associated with that memory (pre-test or baseline).

Once the initial assessment had been completed, participants were separated according to their group assignment. The experimental group received the PASER protocol (a technique with an approximate duration of 15 mins). The control group, in contrast, performed the distractor task, designed to match the duration of the intervention without generating active emotional processing of the memory. The duration of both conditions was equivalent (approximately 15 mins).

Following completion of the intervention or distractor task, the APEEM was administered again to all participants (post-test), assessing the same memory chosen at the beginning.

### Results

In order to evaluate the effect of the PASER technique on emotional disturbance, a 2 × 2 mixed factorial analysis of variance (ANOVA) was conducted, considering the between-subjects factor Intervention Condition (PASER vs. control) and the within-subjects factor Time (pre-test vs. post-test), with the APEEM score as the dependent variable.

Prior to the analysis, compliance with the assumptions was verified. Levene's test for equality of variances was non-significant for both the pre-test measurements [*F*_(1, 46)_ = 2.43, *p* = 0.126] and the post-test measurements [*F*_(1, 46)_ = 2.80, *p* = 0.101], confirming homogeneity of variances between groups. Given that the within-subjects factor has only two levels, the assumption of sphericity is mathematically satisfied, and therefore no correction of the degrees of freedom was required.

The results of the mixed ANOVA indicated:

**Main effect of time:** A significant reduction in emotional disturbance from pre-test to post-test was observed when considering both groups together, *F*_(1, 46)_ = 88.82, *p* < 0.001, and *ηp*^2^ = 0.659. This large effect reflects, in part, the desensitization expected from repeated exposure to the stressful memory, an effect present in both conditions.

**Main effect of intervention condition:** The difference between the PASER group and the Control group, globally averaged across both time points (pre-test and post-test), did not reach statistical significance, *F*_(1, 46)_ = 1.95, *p* = 0.170, and *ηp*^2^ = 0.041. This result is expected given the initial equivalence in emotional disturbance between the experimental and control groups due to random assignment, as well as the baseline measurement, at which neither the PASER treatment nor the control task had yet been administered, which attenuates the differences.

**Condition** **×** **time interaction:** The analysis revealed a significant and robust interaction effect between the Time and Group (treatment) variables, *F*_(1, 46)_ = 15.89, *p* < 0.001, and *ηp*^2^ = 0.257. This result indicates that the pattern of change in emotional disturbance between pre-test and post-test differed significantly according to the assigned condition, with a reduction effect in emotional disturbance being observed among participants who underwent the PASER treatment. The analysis of simple effects allows for a better understanding of the interaction effects, showing that the Intervention Condition variable did not present significant differences under the pre-test (baseline) condition of the Time variable, *t*_(46)_ = 0.321, *p* = 0.75. However, when the simple effect of the Intervention Condition variable was analyzed under the post-test condition of the Time variable, the group that underwent PASER showed a statistically significant reduction in emotional disturbance levels compared with the group that underwent the distractor task (control), *t*_(46)_ = 2.868, *p* < 0.006 (MControl = 28.05; MPASER = 17.62).

**Figure 3:**
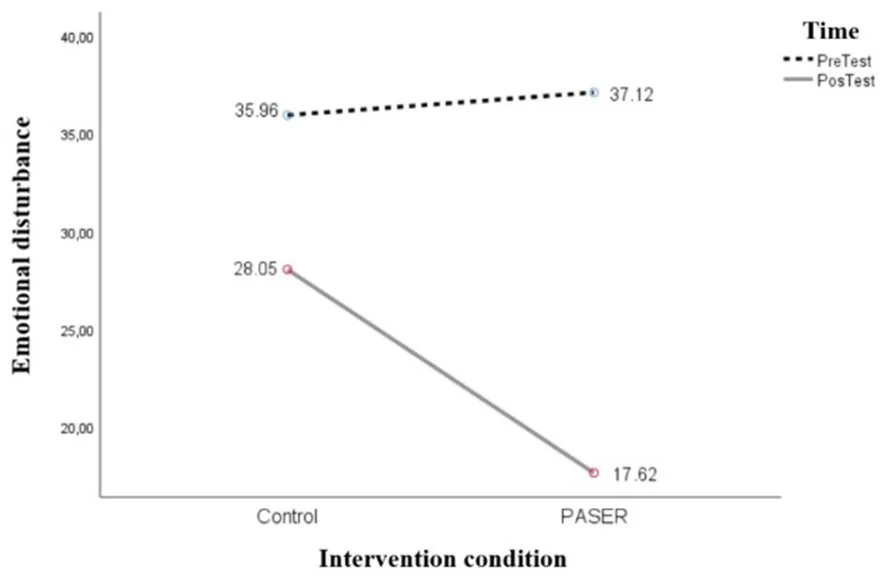
Interaction between time and intervention condition.

As can be seen in Table 2, the experimental group (PASER) showed a marked reduction in APEEM scores (Mpre-test = 37.12; Mpost-test = 17.62), whereas the control group showed a substantially smaller reduction (Mpre-test = 35.96; Mpost-test = 28.05). This difference in the magnitude of change, visually represented in Figure 3 showing the profile plot of estimated marginal means, constitutes the central evidence supporting the study hypothesis: the application of PASER produces a reduction in emotional disturbance that is significantly greater than that attributable to the mere passage of time.

**Table 2:** Estimated marginal means of emotional disturbance (APEEM) by group and measurement time point.

Group	Measurement time point	*M*	SD
Control (*n* = 22)	Pre-test	35.96	12.32
	Post-test	28.05	12.42
PASER (*n* = 26)	Pre-test	37.12	11.34
	Post-test	17.62	11.40

M, mean; SD, standard deviation. Values correspond to the estimated marginal means.

Additionally, the variable Sex was incorporated into the factorial design through a 2 × 2 × 2 ANOVA. No differences were found between men and women, nor were any interaction effects observed. Given the sample size and its imbalance, findings regarding this latter variable should be interpreted with caution (Tables 3–5).

**Table 3:** Descriptive statistics (Study 2).

Measurement time	Intervention condition	Sex	Mean	Standard deviation	*N*
Total pre	CONTROL (distractor task)	Woman	37.0526	13.55021	19
		Man	29.0000	15.87451	3
		Total	35.9545	13.76150	22
	PASER	Woman	37.9524	11.79608	21
		Man	33.6000	9.07193	5
		Total	37.1154	11.29363	26
	Total	Woman	37.5250	12.50228	40
		Man	31.8750	11.16676	8
		Total	36.5833	12.36129	48
Total post	CONTROL (distractor task)	Woman	28.7368	15.37257	19
		Man	23.6667	11.84624	3
		Total	28.0455	14.80179	22
	PASER	Woman	17.8571	10.77166	21
		Man	16.6000	8.96103	5
		Total	17.6154	10.29204	26
	Total	Woman	23.0250	14.10126	40
		Man	19.2500	9.96781	8
		Total	22.3958	13.48402	48

**Table 4:** Tests of between-subjects effects.

Transformed variable: Average								
Origin	Type III sum of squares	df	Mean square	*F*	Sig. (*p*-value)	Partial eta squared	Non-centrality parameter	Observed power^a^
Intersection	39,761.766	1	39,761.766	148.140	0.000	0.771	148.140	1.000
Intervention condition	122.256	1	122.256	0.455	0.503	0.010	0.455	0.101
Sex	276.917	1	276.917	1.032	0.315	0.023	1.032	0.169
Intervention condition by sex	44.548	1	44.548	0.166	0.686	0.004	0.166	0.068
Error	11,809.931	44	268.408					

^*a*^It was calculated using an alpha level of 0.05. The asterisk was used to indicate the interaction between factors.

**Table 5:** Tests of within-subjects contrasts.

Origin	Time	Type III sum of squares	df	Mean square	*F*	Sig. (*p*-value)	Partial eta squared	Non-centrality parameter	Observed power^a^
Time	Linear	2,032.083	1	2,032.083	39.091	0.000	0.470	39.091	1.000
Time by condition	Linear	433.818	1	433.818	8.345	0.006	0.159	8.345	0.807
Time by sex	Linear	29.150	1	29.150	0.561	0.458	0.013	0.561	0.113
Time by condition by sex	Linear	0.010	1	0.010	0.000	0.989	0.000	0.000	0.050
Error (Time)	Linear	2,287.291	44	51.984					

^*a*^It was calculated using an alpha level of 0.05.

### Discussion (Study 2)

The findings of this second experimental study provide robust empirical evidence regarding the effectiveness of the PASER technique for reducing emotional disturbance. By transitioning from a quasi-experimental design (Study 1) to a 2 × 2 mixed factorial design with random assignment, we were able to control confounding variables that limited internal validity.

The central finding of this experiment lies in the significance and robustness of the interaction between Intervention Condition and Time [*F*_(1, 46)_ = 15.89, *p* < 0.001, and *ηp*^2^ = 0.257]. This interaction effect clearly indicates that the gradient of emotional change differs substantially depending on the intervention received. While the control group (subjected to a visual distractor task designed to equate temporal demand) showed an attenuated reduction in APEEM scores, the experimental group exposed to the PASER protocol exhibited a larger and clinically relevant reduction in emotional disturbance levels (Mpre-test = 37.12 vs. Mpost-test = 17.62). The decomposition of this interaction through simple effects analysis confirmed that, although both groups started from statistically equivalent baseline conditions (*p* = 0.75), the post-intervention difference clearly favored PASER [*t*_(46)_ = 2.868, *p* < 0.006], supporting the specificity of the protocol.

Nevertheless, from a strictly methodological perspective, it is essential to examine the behavior of the main effects. The main effect of Time was the largest in the entire model [*F*_(1, 46)_ = 88.82, *p* < 0.001, and *ηp*^2^ = 0.659]. As theoretically expected, this phenomenon captures natural desensitization due to repeated exposure: the simple act of evoking and reactivating a stressful memory at two close time points generates a marginal habituation effect, observable even in the control group (Mpre-test = 35.96 vs. Mpost-test = 28.05). The fact that the main effect of Intervention Condition was not significant (*p* = 0.170) does not invalidate the findings; on the contrary, methodologically it reflects the proper randomization of the design, as uniform baseline measurements reduce global between-subject variance when averaged across both time points.

In conclusion, the overall trajectory of reduction in emotional disturbance observed in the control group (Mpre-test = 35.96 vs. Mpost-test = 28.05) highlights that the mere passage of time between measurements is not a passive factor, but rather a context in which multiple psychophysiological and learning-related processes converge. On one hand, time allows for the homeostatic dissipation of the initial alarm response, while the predictability of the procedure and habituation to the instrument reduce anticipatory anxiety; after having already experienced the structure of the items in the post-test, participants show greater expectations of self-efficacy and perceived control over the assessment situation.

It is important to highlight that the administration of the pre-test itself requires participants to formally engage in an initial structured evocation of the stressful memory for baseline assessment; this initial contact with the stimulus functions normatively as a baseline imaginal exposure that promotes an initial systematic desensitization, prior to any intervention. On the other hand, the subsequent time interval introduces the possibility of incidental processing through potential intrusive thoughts. From emotional processing theories, attentional focus alternation (the back-and-forth flow in which the memory enters and leaves consciousness in parallel with the assigned task) may bifurcate the adaptive response: if re-contact remains within tolerable neurovegetative activation, attentional oscillation operates as a gradient of fragmented desensitization; conversely, if intrusion produces a sharp increase in arousal and the subject abruptly returns to the distractor task using it as cognitive avoidance, a truncated exposure occurs that may promote an opposite sensitization effect.

It should be noted that, although desensitization through formal and incidental exposure is the predominant mechanism at the population level, detailed inspection of individual responses within the data matrix reveals the coexistence of minor and isolated sensitization trajectories, where specific subjects increase their post-test disturbance. Although these atypical responses are methodologically obscured by the aggregation of central tendency measures in group-level analyses, their empirical presence underscores the non-linear nature of stress processing.

The methodological relevance of our findings lies in the fact that, while the control group is left exposed to the stochastic drift of these attentional fluctuations and micro-processes over time, the application of the PASER protocol intervenes by optimizing, channeling, and systematically stabilizing these same neurocognitive mechanisms, achieving a significantly greater (Mpost-test = 17.62) and robust reduction in the desensitization curve.

Finally, the inclusion of the “Sex” variable in the extended 2 × 2 × 2 model did not yield significant differences nor interaction effects with the treatment (*p* > 0.05). However, this result should be interpreted with appropriate caution due to the uneven sex distribution (83.3% women vs. 16.7% men), which reduced the statistical power to detect effects of this variable.

## Conclusion

The findings of the present research suggest that the PASER technique reduces the emotional disturbance associated with recent stressful memories more rapidly than imaginal exposure alone. These results support the potential usefulness of PASER as a brief intervention aimed at facilitating the adaptive processing of stressful experiences and reducing the emotional impact associated with their recall.

From a theoretical perspective, the observed effects are consistent with models of memory reconsolidation, which propose that recently activated memories may be modified through the incorporation of new information and meanings. However, the mechanisms underlying the observed reduction in emotional disturbance cannot be conclusively determined from the present studies and require further investigation with longitudinal designs.

Therefore, the results should be interpreted with caution. Future studies employing larger samples, a more balanced gender distribution, and longitudinal follow-up assessments are needed to replicate and extend these findings, clarify the underlying mechanisms, and evaluate the stability of the observed changes over time. It would also be valuable to analyze the differential effects of PASER in subpopulations with varying levels of emotional vulnerability. Still, the findings do suggest that PASER may represent a promising tool for early psychological intervention in contexts involving recent stressful experiences.

## Data Availability

The original contributions presented in the study are included in the article/Supplementary material, further inquiries can be directed to the corresponding author/s.
