# Supplementary material for: Disrupting emotional memory: PASER as a potential reconsolidation-based intervention in stressful experiences
Source: Front Cognit. 2026 Aug 7;5:1868072. doi: 10.3389/fcogn.2026.1868072 (PMC13493270; doi:10.3389/fcogn.2026.1868072)
Supplement: Supplementary file 1 [file Data_Sheet_1.pdf]

```
 >Warning # 849 in column 23.  Text: es_41
>The LOCALE subcommand of the SET command has an invalid parameter.  It cou
ld
>not be mapped to a valid backend locale.
GET
  FILE='D:\Downloads\Matriz AT UBA 2025.sav'.
DATASET NAME ConjuntoDatos1 WINDOW=FRONT.
DATASET ACTIVATE ConjuntoDatos1.

SAVE OUTFILE='D:\Downloads\Matriz AT UBA 2025.sav'
  /COMPRESSED.
GLM APEEM_1_total APEEM_2_total APEEM_3_total APEEM_4_total
  /WSFACTOR=Tiempo 4 Polynomial
  /METHOD=SSTYPE(3)
  /PLOT=PROFILE(Tiempo) TYPE=BAR ERRORBAR=CI MEANREFERENCE=NO
  /EMMEANS=TABLES(OVERALL)
  /EMMEANS=TABLES(Tiempo) COMPARE ADJ(SIDAK)
  /PRINT=DESCRIPTIVE ETASQ OPOWER HOMOGENEITY
  /CRITERIA=ALPHA(.05)
  /WSDESIGN=Tiempo.
```

Modelo lineal general

Notas

Salida creada		03-JUL-2025 10:40:26
Comentarios		
Entrada	Datos	D:\Downloads\Matriz AT UBA 2025.sav
	Conjunto de datos activo	ConjuntoDatos1
	Filtro	<ninguno>
	Ponderación	<ninguno>
	Segmentar archivo	<ninguno>
	N de filas en el archivo de datos de trabajo	67
Manejo de valores perdidos	Definición de perdidos	Los valores perdidos definidos por el usuario se tratan como perdidos.
	Casos utilizados	Las estadísticas se basan en todos los casos con datos válidos para todas las variables del modelo.
Sintaxis		GLM APEEM_1_total APEEM_2_total APEEM_3_total APEEM_4_total<br>/WSFACTOR=Tiempo 4 Polynomial<br>/METHOD=SSTYPE(3)<br>/PLOT=PROFILE (Tiempo) TYPE=BAR ERRORBAR=CI MEANREFERENCE=NO<br>/EMMEANS=TABLES (OVERALL)<br>/EMMEANS=TABLES (Tiempo) COMPARE ADJ (SIDAK)<br>/PRINT=DESCRIPTIVE ETASQ OPOWER HOMOGENEITY<br>/CRITERIA=ALPHA(.05)<br>/WSDESIGN=Tiempo.
Recursos	Tiempo de procesador	00:00:01,27
	Tiempo transcurrido	00:00:00,79

```
[ConjuntoDatos1] D:\Downloads\Matriz AT UBA 2025.sav
```

Advertencias

La especificación HOMOGENEITY en el subcomando PRINT se ignorará porque no hay factores inter-sujetos.

Factores intra-sujetos

Medida: MEASURE_1

Tiempo	Variable dependiente
1	APEEM_1_total
2	APEEM_2_total
3	APEEM_3_total
4	APEEM_4_total

Estadísticos descriptivos

	Media	Desv. Desviación	N
APEEM_1_total	38,88	13,808	67
APEEM_2_total	36,37	15,185	67
APEEM_3_total	31,36	15,324	67
APEEM_4_total	17,96	11,729	67

Pruebas multivariante[a]

Efecto		Valor	F	gl de hipótesis	gl de error	Sig.
Tiempo	Traza de Pillai	,803	86,685[b]	3,000	64,000	,000
	Lambda de Wilks	,197	86,685[b]	3,000	64,000	,000
	Traza de Hotelling	4,063	86,685[b]	3,000	64,000	,000
	Raíz mayor de Roy	4,063	86,685[b]	3,000	64,000	,000

Pruebas multivariante[a]

Efecto		Eta parcial al cuadrado	Parámetro sin centralidad	Potencia observada[c]
Tiempo	Traza de Pillai	,803	260,054	1,000
	Lambda de Wilks	,803	260,054	1,000
	Traza de Hotelling	,803	260,054	1,000
	Raíz mayor de Roy	,803	260,054	1,000

a. Diseño : Intersección
Diseño intra-sujetos: Tiempo

b. Estadístico exacto

c. Se ha calculado utilizando alpha = .05

Prueba de esfericidad de Mauchly[a]

Medida: MEASURE_1

					Épsilon[b]
Efecto intra-sujetos	W de Mauchly	Aprox. Chi-cuadrado	gl	Sig.	Greenhouse-Geisser
Tiempo	,610	31,973	5	,000	,771

Prueba de esfericidad de Mauchly[a]

Medida: MEASURE_1

	Épsilon[b]	
Efecto intra-sujetos	Huynh-Feldt	Límite inferior
Tiempo	,801	,333

Prueba la hipótesis nula de que la matriz de covarianzas de error de las variables dependientes con transformación ortonormalizada es proporcional a una matriz de identidad.

a. Diseño : Intersección
Diseño intra-sujetos: Tiempo

b. Se puede utilizar para ajustar los grados de libertad para las pruebas promedio de significación. Las pruebas corregidas se visualizan en la tabla de pruebas de efectos intra-sujetos.

Pruebas de efectos intra-sujetos

Medida: MEASURE_1

Origen		Tipo III de suma de cuadrados	gl	Media cuadrática	F
Tiempo	Esfericidad asumida	17499,627	3	5833,209	149,272
	Greenhouse-Geisser	17499,627	2,313	7565,979	149,272
	Huynh-Feldt	17499,627	2,402	7285,898	149,272
	Límite inferior	17499,627	1,000	17499,627	149,272
Error(Tiempo)	Esfericidad asumida	7737,373	198	39,078	
	Greenhouse-Geisser	7737,373	152,654	50,686	
	Huynh-Feldt	7737,373	158,522	48,809	
	Límite inferior	7737,373	66,000	117,233	

Pruebas de efectos intra-sujetos

Medida: MEASURE_1

Origen		Sig.	Eta parcial al cuadrado	Parámetro sin centralidad	Potencia observada[a]
Tiempo	Esfericidad asumida	,000	,693	447,817	1,000
	Greenhouse-Geisser	,000	,693	345,257	1,000
	Huynh-Feldt	,000	,693	358,530	1,000
	Límite inferior	,000	,693	149,272	1,000
Error(Tiempo)	Esfericidad asumida				
	Greenhouse-Geisser				
	Huynh-Feldt				
	Límite inferior				

a. Se ha calculado utilizando alpha = .05

Pruebas de contrastes intra-sujetos

Medida: MEASURE_1

Origen	Tiempo	Tipo III de suma de cuadrados	gl	Media cuadrática	F	Sig.
Tiempo	Lineal	15395,346	1	15395,346	243,233	,000
	Cuadrático	1988,433	1	1988,433	56,190	,000
	Cúbico	115,848	1	115,848	6,245	,015
Error(Tiempo)	Lineal	4177,454	66	63,295		
	Cuadrático	2335,567	66	35,387		
	Cúbico	1224,352	66	18,551		

Pruebas de contrastes intra-sujetos

Medida: MEASURE_1

Origen	Tiempo	Eta parcial al cuadrado	Parámetro sin centralidad	Potencia observada[a]
Tiempo	Lineal	,787	243,233	1,000
	Cuadrático	,460	56,190	1,000
	Cúbico	,086	6,245	,692
Error(Tiempo)	Lineal			
	Cuadrático			
	Cúbico			

a. Se ha calculado utilizando alpha = .05

Pruebas de efectos inter-sujetos

Medida: MEASURE_1

Variable transformada: Promedio

Origen	Tipo III de suma de cuadrados	gl	Media cuadrática	F	Sig.	Eta parcial al cuadrado
Intersección	259909,388	1	259909,388	384,261	,000	,853
Error	44641,612	66	676,388			

Pruebas de efectos inter-sujetos

Medida: MEASURE_1

Variable transformada: Promedio

Origen	Parámetro sin centralidad	Potencia observada[a]
Intersección	384,261	1,000
Error		

a. Se ha calculado utilizando alpha = .05

Medias marginales estimadas

1. Media global

Medida: MEASURE_1

Media	Desv. Error	Intervalo de confianza al 95%	
		Límite inferior	Límite superior
31,142	1,589	27,970	34,314

2. Tiempo

Estimaciones

Medida: MEASURE_1

Tiempo	Media	Desv. Error	Intervalo de confianza al 95%	
			Límite inferior	Límite superior
1	38,881	1,687	35,513	42,249
2	36,373	1,855	32,669	40,077
3	31,358	1,872	27,620	35,096
4	17,955	1,433	15,094	20,816

Comparaciones por parejas

Medida: MEASURE_1

(I) Tiempo	(J) Tiempo	Diferencia de medias (I-J)	Desv. Error	Sig.[b]	95% de intervalo de confianza para diferencia[b] Límite inferior	Límite superior
1	2	2,507*	,774	,011	,408	4,607
	3	7,522*	1,073	,000	4,612	10,433
	4	20,925*	1,303	,000	17,392	24,459
2	1	-2,507*	,774	,011	-4,607	-,408
	3	5,015*	,864	,000	2,672	7,358
	4	18,418*	1,267	,000	14,981	21,855
3	1	-7,522*	1,073	,000	-10,433	-4,612
	2	-5,015*	,864	,000	-7,358	-2,672
	4	13,403*	1,095	,000	10,432	16,373
4	1	-20,925*	1,303	,000	-24,459	-17,392
	2	-18,418*	1,267	,000	-21,855	-14,981
	3	-13,403*	1,095	,000	-16,373	-10,432

Se basa en medias marginales estimadas

*. La diferencia de medias es significativa en el nivel .05.

b. Ajuste para varias comparaciones: Sidak.

Pruebas multivariante

	Valor	F	gl de hipótesis	gl de error	Sig.	Eta parcial al cuadrado
Traza de Pillai	,803	86,685[a]	3,000	64,000	,000	,803
Lambda de Wilks	,197	86,685[a]	3,000	64,000	,000	,803
Traza de Hotelling	4,063	86,685[a]	3,000	64,000	,000	,803
Raíz mayor de Roy	4,063	86,685[a]	3,000	64,000	,000	,803

Pruebas multivariante

	Parámetro sin centralidad	Potencia observada[b]
Traza de Pillai	260,054	1,000
Lambda de Wilks	260,054	1,000
Traza de Hotelling	260,054	1,000
Raíz mayor de Roy	260,054	1,000

Cada F prueba el efecto multivariante de Tiempo. Estas pruebas se basan en las comparaciones por parejas linealmente independientes entre las medias marginales estimadas.

a. Estadístico exacto

b. Se ha calculado utilizando alpha = .05

Gráficos de perfil

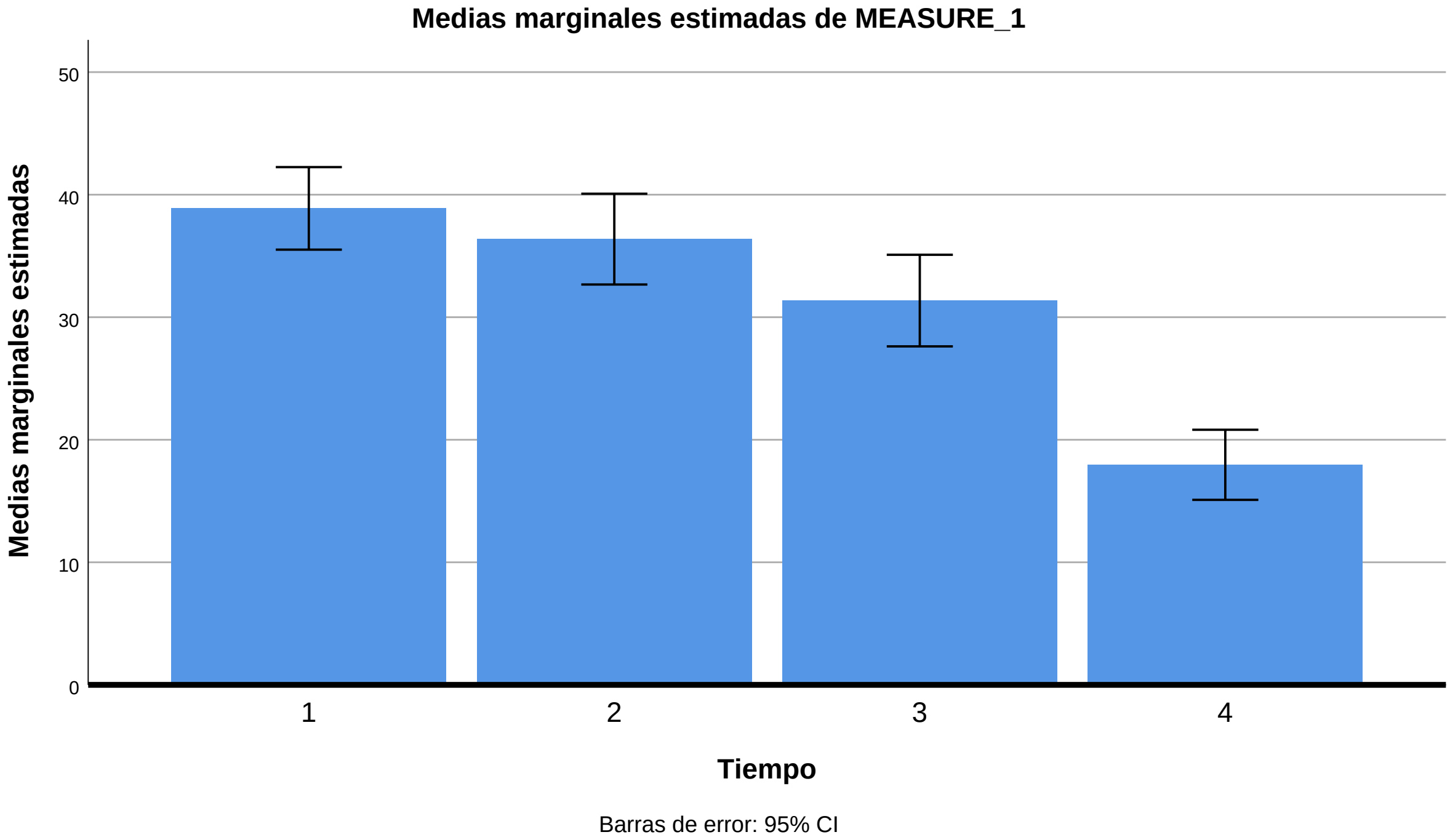


```
GLM Cambio1_EfectoClase1 Cambio2_EfectoClase2 Cambio3_EfectoProtocolo
 /WSFACTOR=Tiempo 3 Polynomial
 /METHOD=SSTYPE(3)
 /PLOT=PROFILE(Tiempo) TYPE=BAR ERRORBAR=CI MEANREFERENCE=NO
 /EMMEANS=TABLES(OVERALL)
 /EMMEANS=TABLES(Tiempo) COMPARE ADJ(BONFERRONI)
 /PRINT=DESCRIPTIVE ETASQ OPOWER
 /CRITERIA=ALPHA(.05)
 /WSDESIGN=Tiempo.
```

Modelo lineal general

Notas

Salida creada		03-JUL-2025 10:58:11
Comentarios		
Entrada	Datos	D:\Downloads\Matriz AT UBA 2025.sav
	Conjunto de datos activo	ConjuntoDatos1
	Filtro	<ninguno>
	Ponderación	<ninguno>
	Segmentar archivo	<ninguno>
	N de filas en el archivo de datos de trabajo	67
Manejo de valores perdidos	Definición de perdidos	Los valores perdidos definidos por el usuario se tratan como perdidos.
	Casos utilizados	Las estadísticas se basan en todos los casos con datos válidos para todas las variables del modelo.
Sintaxis		GLM Cambio1_EfectoClase1 Cambio2_EfectoClase2 Cambio3_EfectoProtocolo /WSFACTOR=Tiempo 3 Polynomial /METHOD=SSTYPE(3) /PLOT=PROFILE (Tiempo) TYPE=BAR ERRORBAR=CI MEANREFERENCE=NO /EMMEANS=TABLES (OVERALL) /EMMEANS=TABLES (Tiempo) COMPARE ADJ (BONFERRONI) /PRINT=DESCRIPTIVE ETASQ OPOWER /CRITERIA=ALPHA(.05) /WSDESIGN=Tiempo.
Recursos	Tiempo de procesador	00:00:00,59
	Tiempo transcurrido	00:00:00,16

Factores intra-sujetos

Medida: MEASURE_1

Tiempo	Variable dependiente
1	Cambio1_EfectoClase1
2	Cambio2_EfectoClase2
3	Cambio3_EfectoProtocolo

Estadísticos descriptivos

	Media	Desv. Desviación	N
Cambio1_EfectoClase1	-2,5075	6,33741	67
Cambio2_EfectoClase2	-5,0149	7,06998	67
Cambio3_EfectoProtocolo	-13,4030	8,96468	67

Pruebas multivariante[a]

Efecto		Valor	F	gl de hipótesis	gl de error	Sig.
Tiempo	Traza de Pillai	,463	28,056[b]	2,000	65,000	,000
	Lambda de Wilks	,537	28,056[b]	2,000	65,000	,000
	Traza de Hotelling	,863	28,056[b]	2,000	65,000	,000
	Raíz mayor de Roy	,863	28,056[b]	2,000	65,000	,000

Pruebas multivariante[a]

Efecto		Eta parcial al cuadrado	Parámetro sin centralidad	Potencia observada[c]
Tiempo	Traza de Pillai	,463	56,113	1,000
	Lambda de Wilks	,463	56,113	1,000
	Traza de Hotelling	,463	56,113	1,000
	Raíz mayor de Roy	,463	56,113	1,000

a. Diseño : Intersección
Diseño intra-sujetos: Tiempo

b. Estadístico exacto

c. Se ha calculado utilizando alpha = .05

Prueba de esfericidad de Mauchly[a]

Medida: MEASURE_1

Efecto intra-sujetos	W de Mauchly	Aprox. Chi-cuadrado	gl	Sig.	Épsilon[b] Greenhouse-Geisser
Tiempo	,948	3,461	2	,177	,951

Prueba de esfericidad de Mauchly[a]

Medida: MEASURE_1

Efecto intra-sujetos	Épsilon[b] Huynh-Feldt	Límite inferior
Tiempo	,978	,500

Prueba la hipótesis nula de que la matriz de covarianzas de error de las variables dependientes con transformación ortonormalizada es proporcional a una matriz de identidad.

a. Diseño : Intersección
Diseño intra-sujetos: Tiempo

b. Se puede utilizar para ajustar los grados de libertad para las pruebas promedio de significación. Las pruebas corregidas se visualizan en la tabla de pruebas de efectos intra-sujetos.

Pruebas de efectos intra-sujetos

Medida: MEASURE_1

Origen		Tipo III de suma de cuadrados	gl	Media cuadrática	F
Tiempo	Esfericidad asumida	4363,025	2	2181,512	32,901
	Greenhouse-Geisser	4363,025	1,901	2294,623	32,901
	Huynh-Feldt	4363,025	1,956	2230,268	32,901
	Límite inferior	4363,025	1,000	4363,025	32,901
Error(Tiempo)	Esfericidad asumida	8752,308	132	66,305	
	Greenhouse-Geisser	8752,308	125,493	69,743	
	Huynh-Feldt	8752,308	129,114	67,787	
	Límite inferior	8752,308	66,000	132,611	

Pruebas de efectos intra-sujetos

Medida: MEASURE_1

Origen		Sig.	Eta parcial al cuadrado	Parámetro sin centralidad	Potencia observada[a]
Tiempo	Esfericidad asumida	,000	,333	65,802	1,000
	Greenhouse-Geisser	,000	,333	62,558	1,000
	Huynh-Feldt	,000	,333	64,363	1,000
	Límite inferior	,000	,333	32,901	1,000
Error(Tiempo)	Esfericidad asumida				
	Greenhouse-Geisser				
	Huynh-Feldt				
	Límite inferior				

a. Se ha calculado utilizando alpha = .05

Pruebas de contrastes intra-sujetos

Medida: MEASURE_1

Origen	Tiempo	Tipo III de suma de cuadrados	gl	Media cuadrática	F	Sig.
Tiempo	Lineal	3976,866	1	3976,866	56,190	,000
	Cuadrático	386,159	1	386,159	6,245	,015
Error(Tiempo)	Lineal	4671,134	66	70,775		
	Cuadrático	4081,174	66	61,836		

Pruebas de contrastes intra-sujetos

Medida: MEASURE_1

Origen	Tiempo	Eta parcial al cuadrado	Parámetro sin centralidad	Potencia observada[a]
Tiempo	Lineal	,460	56,190	1,000
	Cuadrático	,086	6,245	,692
Error(Tiempo)	Lineal			
	Cuadrático			

a. Se ha calculado utilizando alpha = .05

Pruebas de efectos inter-sujetos

Medida: MEASURE_1

Variable transformada: Promedio

Origen	Tipo III de suma de cuadrados	gl	Media cuadrática	F	Sig.	Eta parcial al cuadrado
Intersección	9779,124	1	9779,124	258,010	,000	,796
Error	2501,542	66	37,902			

Pruebas de efectos inter-sujetos

Medida: MEASURE_1

Variable transformada: Promedio

Origen	Parámetro sin centralidad	Potencia observada[a]
Intersección	258,010	1,000
Error		

a. Se ha calculado utilizando alpha = .05

Medias marginales estimadas

1. Media global

Medida: MEASURE_1

Media	Desv. Error	Intervalo de confianza al 95%	
		Límite inferior	Límite superior
-6,975	,434	-7,842	-6,108

2. Tiempo

Estimaciones

Medida: MEASURE_1

Tiempo	Media	Desv. Error	Intervalo de confianza al 95%	
			Límite inferior	Límite superior
1	-2,507	,774	-4,053	-,962
2	-5,015	,864	-6,739	-3,290
3	-13,403	1,095	-15,590	-11,216

Comparaciones por parejas

Medida: MEASURE_1

(I) Tiempo	(J) Tiempo	Diferencia de medias (I-J)	Desv. Error	Sig.[b]	95% de intervalo de confianza para diferencia[b] Límite inferior	Límite superior
1	2	2,507	1,241	,142	-,541	5,556
	3	10,896[*]	1,454	,000	7,325	14,466
2	1	-2,507	1,241	,142	-5,556	,541
	3	8,388[*]	1,512	,000	4,674	12,102
3	1	-10,896[*]	1,454	,000	-14,466	-7,325
	2	-8,388[*]	1,512	,000	-12,102	-4,674

Se basa en medias marginales estimadas

*. La diferencia de medias es significativa en el nivel .05.

b. Ajuste para varias comparaciones: Bonferroni.

Pruebas multivariante

	Valor	F	gl de hipótesis	gl de error	Sig.	Eta parcial al cuadrado
Traza de Pillai	,463	28,056[a]	2,000	65,000	,000	,463
Lambda de Wilks	,537	28,056[a]	2,000	65,000	,000	,463
Traza de Hotelling	,863	28,056[a]	2,000	65,000	,000	,463
Raíz mayor de Roy	,863	28,056[a]	2,000	65,000	,000	,463

Pruebas multivariante

	Parámetro sin centralidad	Potencia observada[b]
Traza de Pillai	56,113	1,000
Lambda de Wilks	56,113	1,000
Traza de Hotelling	56,113	1,000
Raíz mayor de Roy	56,113	1,000

Cada F prueba el efecto multivariante de Tiempo. Estas pruebas se basan en las comparaciones por parejas linealmente independientes entre las medias marginales estimadas.

a. Estadístico exacto

b. Se ha calculado utilizando alpha = .05

Gráficos de perfil

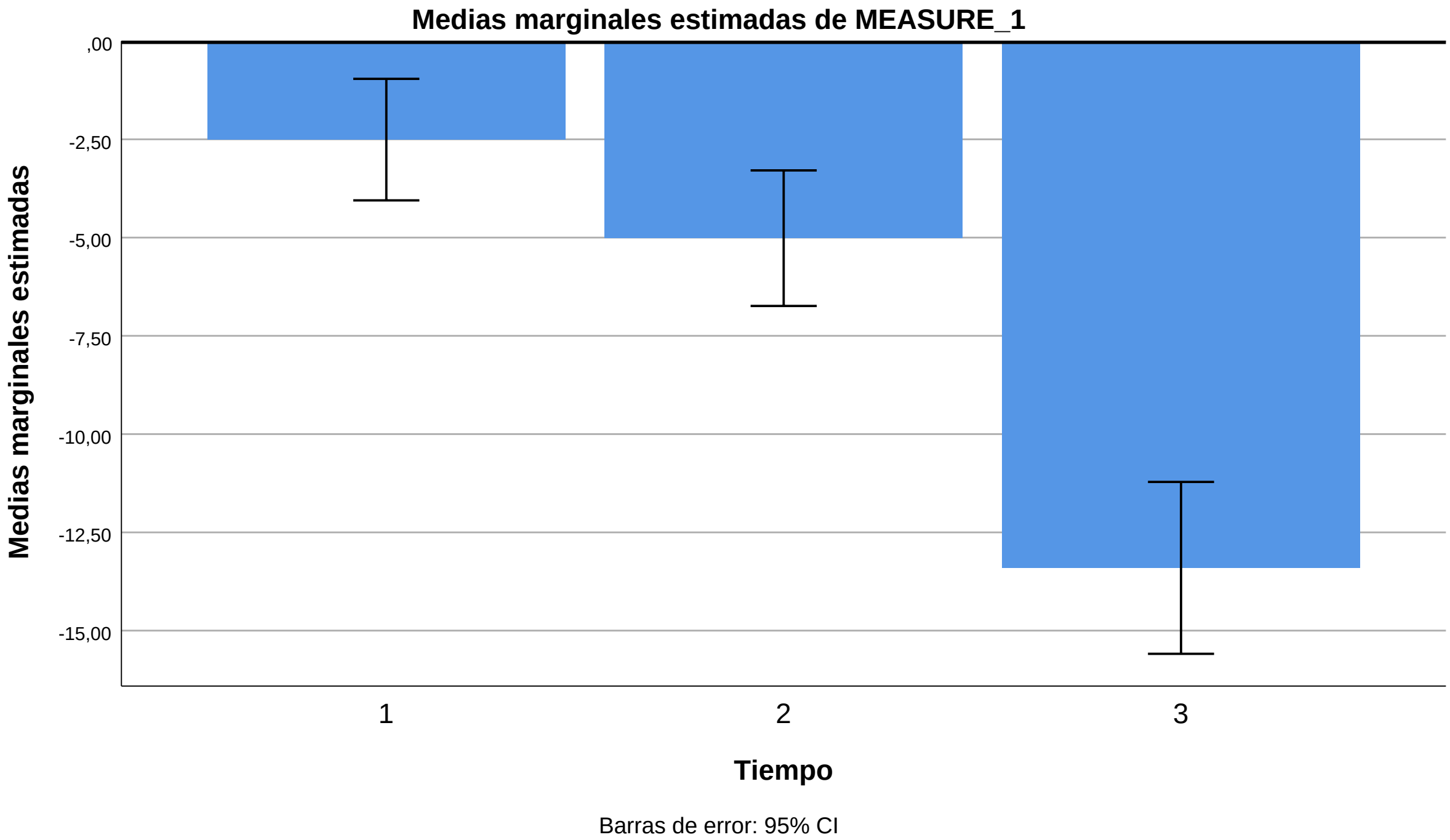
Medias marginales estimadas de MEASURE_1
Medias marginales estimadas
,00
-2,50
-5,00
-7,50
-10,00
-12,50
-15,00
1
2
3
Tiempo
Barras de error: 95% CI
