# Supplementary material for: Disrupting emotional memory: PASER as a potential reconsolidation-based intervention in stressful experiences
Source: Front Cognit. 2026 Aug 7;5:1868072. doi: 10.3389/fcogn.2026.1868072 (PMC13493270; doi:10.3389/fcogn.2026.1868072)
Supplement: Supplementary file 2 [file Data_Sheet_2.docx]

Descriptivos

| **Estadísticos descriptivos** | | | | | | |
| --- | --- | --- | --- | --- | --- | --- |
|  | N | Mínimo | Máximo | Media | Desv. Desviación | Varianza |
| EDAD | 48 | 19 | 35 | 23,92 | 3,024 | 9,142 |
| N válido (por lista) | 48 |  |  |  |  |  |

| **SEXO** | | | | | |
| --- | --- | --- | --- | --- | --- |
|  | | Frecuencia | Porcentaje | Porcentaje válido | Porcentaje acumulado |
| Válido | 1 | 40 | 83,3 | 83,3 | 83,3 |
|  | 2 | 8 | 16,7 | 16,7 | 100,0 |
|  | Total | 48 | 100,0 | 100,0 |  |

**ANOVA MIXTO 2 x 2**

| **Factores intra-sujetos** | |
| --- | --- |
| Medida: MEASURE_1 | |
| Tiempo | Variable dependiente |
| 1 | TOTALPRE1_A |
| 2 | TOTALPOST1_A |

| **Factores inter-sujetos** | | |
| --- | --- | --- |
|  | | N |
| GRUPO | 0 | 22 |
|  | 1 | 26 |

| **Estadísticos descriptivos** | | | | |
| --- | --- | --- | --- | --- |
|  | GRUPO | Media | Desv. Desviación | N |
| TOTAL PRE | 0 | 35,9545 | 13,76150 | 22 |
|  | 1 | 37,1154 | 11,29363 | 26 |
|  | Total | 36,5833 | 12,36129 | 48 |
| TOTAL POST | 0 | 28,0455 | 14,80179 | 22 |
|  | 1 | 17,6154 | 10,29204 | 26 |
|  | Total | 22,3958 | 13,48402 | 48 |

| **Pruebas de contrastes intra-sujetos** | | | | | | | | | |
| --- | --- | --- | --- | --- | --- | --- | --- | --- | --- |
| Medida: MEASURE_1 | | | | | | | | | |
| Origen | Tiempo | Tipo III de suma de cuadrados | gl | Media cuadrática | F | Sig. | Eta parcial al cuadrado | Parámetro sin centralidad | Potencia observada^a^ |
| Tiempo | Lineal | 4476,247 | 1 | 4476,247 | 88,824 | ,000 | ,659 | 88,824 | 1,000 |
| Tiempo * Condición de Intervención | Lineal | 800,497 | 1 | 800,497 | 15,885 | ,000 | ,257 | 15,885 | ,974 |
| Error(Tiempo) | Lineal | 2318,159 | 46 | 50,395 |  |  |  |  |  |
| a. Se ha calculado utilizando alpha = .05 | | | | | | | | | |

| **Prueba de igualdad de Levene de varianzas de error^a^** | | | | | |
| --- | --- | --- | --- | --- | --- |
|  | | Estadístico de Levene | gl1 | gl2 | Sig. |
| TOTAL PRE | Se basa en la media | 2,429 | 1 | 46 | ,126 |
|  | Se basa en la mediana | 2,420 | 1 | 46 | ,127 |
|  | Se basa en la mediana y con gl ajustado | 2,420 | 1 | 45,839 | ,127 |
|  | Se basa en la media recortada | 2,429 | 1 | 46 | ,126 |
| TOTAL POST | Se basa en la media | 2,802 | 1 | 46 | ,101 |
|  | Se basa en la mediana | 2,809 | 1 | 46 | ,101 |
|  | Se basa en la mediana y con gl ajustado | 2,809 | 1 | 40,558 | ,101 |
|  | Se basa en la media recortada | 2,821 | 1 | 46 | ,100 |
| Prueba la hipótesis nula de que la varianza de error de la variable dependiente es igual entre grupos. | | | | | |
| a. Diseño : Intersección + GRUPO  Diseño intra-sujetos: Tiempo | | | | | |

| **Pruebas de efectos inter-sujetos** | | | | | | | | |
| --- | --- | --- | --- | --- | --- | --- | --- | --- |
| Medida: MEASURE_1 | | | | | | | | |
| Variable transformada: Promedio | | | | | | | | |
| Origen | Tipo III de suma de cuadrados | gl | Media cuadrática | F | Sig. | Eta parcial al cuadrado | Parámetro sin centralidad | Potencia observada^a^ |
| Intersección | 83994,599 | 1 | 83994,599 | 319,409 | ,000 | ,874 | 319,409 | 1,000 |
| GRUPO | 511,932 | 1 | 511,932 | 1,947 | ,170 | ,041 | 1,947 | ,277 |
| Error | 12096,558 | 46 | 262,969 |  |  |  |  |  |
| a. Se ha calculado utilizando alpha = .05 | | | | | | | | |

| **Estimaciones de parámetro** | | | | | | | | | | |
| --- | --- | --- | --- | --- | --- | --- | --- | --- | --- | --- |
| Variable dependiente | Parámetro | B | Desv. Error | t | Sig. | Intervalo de confianza al 95% | | Eta parcial al cuadrado | Parámetro sin centralidad | Potencia observada^b^ |
|  |  |  |  |  |  | Límite inferior | Límite superior |  |  |  |
| TOTAL PRE | Intersección | 37,115 | 2,448 | 15,163 | ,000 | 32,188 | 42,042 | ,833 | 15,163 | 1,000 |
|  | [GRUPO=0] | -1,161 | 3,616 | -,321 | ,750 | -8,438 | 6,117 | ,002 | ,321 | ,061 |
|  | [GRUPO=1] | 0^a^ | . | . | . | . | . | . | . | . |
| TOTAL POST | Intersección | 17,615 | 2,462 | 7,155 | ,000 | 12,660 | 22,571 | ,527 | 7,155 | 1,000 |
|  | [GRUPO=0] | 10,430 | 3,637 | 2,868 | ,006 | 3,110 | 17,750 | ,152 | 2,868 | ,802 |
|  | [GRUPO=1] | 0^a^ | . | . | . | . | . | . | . | . |
| a. Este parámetro está establecido en cero porque es redundante. | | | | | | | | | | |
| b. Se ha calculado utilizando alpha = .05 | | | | | | | | | | |

| **4. GRUPO * Tiempo** | | | | | |
| --- | --- | --- | --- | --- | --- |
| Medida: MEASURE_1 | | | | | |
| GRUPO | Tiempo | Media | Desv. Error | Intervalo de confianza al 95% | |
|  |  |  |  | Límite inferior | Límite superior |
| 0 | 1 | 35,955 | 2,661 | 30,598 | 41,311 |
|  | 2 | 28,045 | 2,676 | 22,658 | 33,433 |
| 1 | 1 | 37,115 | 2,448 | 32,188 | 42,042 |
|  | 2 | 17,615 | 2,462 | 12,660 | 22,571 |

**Efectos simples**

**Efecto simple grupo bajo condición de tiempo cero (pre- medida de base)**

| **Estadísticas de grupo** | | | | | |
| --- | --- | --- | --- | --- | --- |
|  | GRUPO | N | Media | Desv. Desviación | Desv. Error promedio |
| TOTAL PRE | 1 | 26 | 37,1154 | 11,29363 | 2,21486 |
|  | 0 | 22 | 35,9545 | 13,76150 | 2,93396 |

| **Prueba de muestras independientes** | | | | | | | | | | |
| --- | --- | --- | --- | --- | --- | --- | --- | --- | --- | --- |
|  | | Prueba de Levene de igualdad de varianzas | | prueba t para la igualdad de medias | | | | | | |
|  |  | F | Sig. | t | gl | Sig. (bilateral) | Diferencia de medias | Diferencia de error estándar | 95% de intervalo de confianza de la diferencia | |
|  |  |  |  |  |  |  |  |  | Inferior | Superior |
| TOTAL PRE | Se asumen varianzas iguales | 2,429 | ,126 | ,321 | 46 | ,750 | 1,16084 | 3,61552 | -6,11682 | 8,43849 |
|  | No se asumen varianzas iguales |  |  | ,316 | 40,662 | ,754 | 1,16084 | 3,67611 | -6,26508 | 8,58676 |

Efecto simple Grupo bajo condición constante de tiempo 10 minutos transcurridos (post)

| **Estadísticas de grupo** | | | | | |
| --- | --- | --- | --- | --- | --- |
|  | GRUPO | N | Media | Desv. Desviación | Desv. Error promedio |
| TOTAL POST | 1 | 26 | 17,6154 | 10,29204 | 2,01844 |
|  | 0 | 22 | 28,0455 | 14,80179 | 3,15575 |

| **Prueba de muestras independientes** | | | | | | | | | | | |
| --- | --- | --- | --- | --- | --- | --- | --- | --- | --- | --- | --- |
|  | | Prueba de Levene de igualdad de varianzas | | prueba t para la igualdad de medias | | | | | | | |
|  |  | F | Sig. | t | gl | Sig. (bilateral) | Diferencia de medias | Diferencia de error estándar | 95% de intervalo de confianza de la diferencia | |  |
|  |  |  |  |  |  |  |  |  | Inferior | Superior |  |
| TOTAL POST | Se asumen varianzas iguales | 2,802 | ,101 | -2,868 | 46 | ,006 | -10,43007 | 3,63652 | -17,75001 | -3,11013 |  |
|  | No se asumen varianzas iguales |  |  | -2,784 | 36,557 | ,008 | -10,43007 | 3,74605 | -18,02338 | -2,83676 |  |

Efecto simple variable tiempo bajo condición sin paser CONTROL

| **Estadísticas de muestras emparejadas** | | | | | |
| --- | --- | --- | --- | --- | --- |
|  | | Media | N | Desv. Desviación | Desv. Error promedio |
| Par 1 | TOTAL PRE | 35,9545 | 22 | 13,76150 | 2,93396 |
|  | TOTAL POST | 28,0455 | 22 | 14,80179 | 3,15575 |

| **Prueba de muestras emparejadas** | | | | | | | | | |
| --- | --- | --- | --- | --- | --- | --- | --- | --- | --- |
|  | | Diferencias emparejadas | | | | | t | gl | Sig. (bilateral) |
|  |  | Media | Desv. Desviación | Desv. Error promedio | 95% de intervalo de confianza de la diferencia | |  |  |  |
|  |  |  |  |  | Inferior | Superior |  |  |  |
| Par 1 | TOTAL PRE - TOTAL POST | 7,90909 | 10,58709 | 2,25718 | 3,21504 | 12,60315 | 3,504 | 21 | ,002 |

Efecto simple variable tiempo bajo condición PASER

| **Estadísticas de muestras emparejadas** | | | | | |
| --- | --- | --- | --- | --- | --- |
|  | | Media | N | Desv. Desviación | Desv. Error promedio |
| Par 1 | TOTAL PRE | 37,1154 | 26 | 11,29363 | 2,21486 |
|  | TOTAL POST | 17,6154 | 26 | 10,29204 | 2,01844 |

| **Prueba de muestras emparejadas** | | | | | | | | | | |
| --- | --- | --- | --- | --- | --- | --- | --- | --- | --- | --- |
|  | | Diferencias emparejadas | | | | | t | gl | Sig. (bilateral) | |
|  |  | Media | Desv. Desviación | Desv. Error promedio | 95% de intervalo de confianza de la diferencia | |  |  |  |  |
|  |  |  |  |  | Inferior | Superior |  |  |  |  |
| Par 1 | TOTAL PRE - TOTAL POST | 19,50000 | 9,55510 | 1,87391 | 15,64061 | 23,35939 | 10,406 | 25 | ,000 |  |
